# Development of a Multi-Channel Piezoelectric Acoustic Sensor Based on an Artificial Basilar Membrane

**DOI:** 10.3390/s140100117

**Published:** 2013-12-20

**Authors:** Youngdo Jung, Jun-Hyuk Kwak, Young Hwa Lee, Wan Doo Kim, Shin Hur

**Affiliations:** Department of Nature-Inspired Nanoconvergence Systems, Korea Institute of Machinery and Materials, 156 Gajeongbuk-ro, Yuseong-gu, Daejeon 304-343, Korea; E-Mails: yjung@kimm.re.kr (Y.J.); jhkwak@kimm.re.kr (J.-H.K.); yhlee12@kimm.re.kr (Y.H.L.); wdkim@kimm.re.kr (W.D.K.)

**Keywords:** cochlea, piezoelectric, microelectromechanical system (MEMS), artificial basilar membrane (ABM), laser Doppler vibrometer (LDV)

## Abstract

In this research, we have developed a multi-channel piezoelectric acoustic sensor (McPAS) that mimics the function of the natural basilar membrane capable of separating incoming acoustic signals mechanically by their frequency and generating corresponding electrical signals. The McPAS operates without an external energy source and signal processing unit with a vibrating piezoelectric thin film membrane. The shape of the vibrating membrane was chosen to be trapezoidal such that different locations of membrane have different local resonance frequencies. The length of the membrane is 28 mm and the width of the membrane varies from 1 mm to 8 mm. Multiphysics finite element analysis (FEA) was carried out to predict and design the mechanical behaviors and piezoelectric response of the McPAS model. The designed McPAS was fabricated with a MEMS fabrication process based on the simulated results. The fabricated device was tested with a mouth simulator to measure its mechanical and piezoelectrical frequency response with a laser Doppler vibrometer and acoustic signal analyzer. The experimental results show that the as fabricated McPAS can successfully separate incoming acoustic signals within the 2.5 kHz–13.5 kHz range and the maximum electrical signal output upon acoustic signal input of 94 dBSPL was 6.33 mVpp. The performance of the fabricated McPAS coincided well with the designed parameters.

## 1. Introduction

The human basilar membrane plays an important role in analyzing the frequencies of incoming soundwaves. Békésy defined the phenomenon of frequency separation studying the structure of the basilar membrane within a cochlea [1]. The basilar membrane has a thin and long breadth and is in the shape of a long trapezoid shape. The base of a basilar membrane that is close to the oval window is thick, with a short breadth giving it high rigidity and the apex is thin with a long breadth giving it high flexibility. Therefore, base area resonates with the higher frequency components of sound waves and the apex area resonates with the lower frequency components. Stereocilia that project from the top of hair cells inside the cochlear are arranged underneath a basilar membrane. As a basilar membrane vibrates, stereocilia come in contact with the basilar membrane and the tip links that are connected between stereocilia are pulled, which in turn opens ion channels and bioelectrical signals are generated and sent to the auditory nerves [2]. According to the World Health Organization, there are near 300 million patients with moderate to profound hearing impairments. Patients having severe hearing loss from damaged cochlea cannot benefit from hearing aids that just amplifies incoming acoustic signals and thus need a cochlear implant to restore their hearing. Conventional cochlear implants consist of a microphone for converting sound into electrical signals, a signal processor for handling the converted electrical signals, an inductive coil for transmitting the processed electrical signal from outside to inside the body, and an electrode array for stimulating nerve cells. The disadvantages of current systems, such as indispensability of extracorporeal devices, small number of electrodes, and frequent recharging requirements caused by large power consumption, have limited their widespread use among the majority of the hearing-impaired [3]. Recently, some researchers have studied the development of next generation cochlear implants that are superior to conventional cochlear implants. Xu *et al.* developed an acoustic sensor by use of an epoxy resonance cantilever structure to mimic the basilar membrane and realized a frequency filter function. The measuring method utilized laser diodes and photosensitive diodes to monitor the intensity of the laser rays [4]. Chen *et al.* developed a beam array fixed on a trapezoid channel and investigated its vibrating characteristics in water. Despite the frequency selectivity of cantilevers or beams, their mechanical strength may not be enough for the implantation as the artificial cochlear for the long period [5]. White and Grosh designed a cochlear-like acoustic sensor based on the Wentzel-Kramer-Brillouin (WKB) approximate technique and fabricated with MEMS techniques [6] and developed a biomimetic acoustic sensor made of a polyimide membrane with Si_3_N_4_ beams based on finite element analysis and measured the behavior of artificial basilar membranes using capacitive sensors [7]. However, their research focused on the simulation and experiments of the acoustic sensor for its mechanical vibration and not for electrical signal conversion. Wittbrodt *et al.* developed a physical cochlear model made of a polyimide membrane with Al beams and measured the behavior of artificial basilar membranes using a laser vibrometer [8,9]. All of the above studies performed studies that mimic the frequency separation functions of the basilar membrane and did not try to mimic the function of ion channels. Shintaku *et al.* developed a biomimetic acoustic sensor that has the functions of frequency separation and conversion of acoustic waves to electrical signals using a piezoelectric membrane structure, but the frequency bandwidth presented was narrow and the electrical signals measured were shown for only a few electrode channels [10].

The objective of this research was to develop a biomimetic acoustic sensor fabricated with a piezoelectric membrane capable of frequency separation and electric signal generation like a human cochlear basilar membrane. Simulation and experiments for both mechanical vibration and piezoelectric signal conversion of the acoustic sensor were carried out. A piezoelectric thin film was processed with microfabrication techniques to form a trapezoid membrane with metal line electrodes. A non-contact laser Doppler vibrometer (LDV) system and acoustic signal analyzer were used in characterizing the vibratory properties and piezoelectric output of the developed multi-channel piezoelectric acoustic sensor (McPAS). Experimental results showed that the McPAS is capable of frequency separation through its locally resonating vibration and conversion of acoustic signals into a locally separated electrical signal output.

## 2. Design and Fabrication

### 2.1. Design

**Figure 1.**
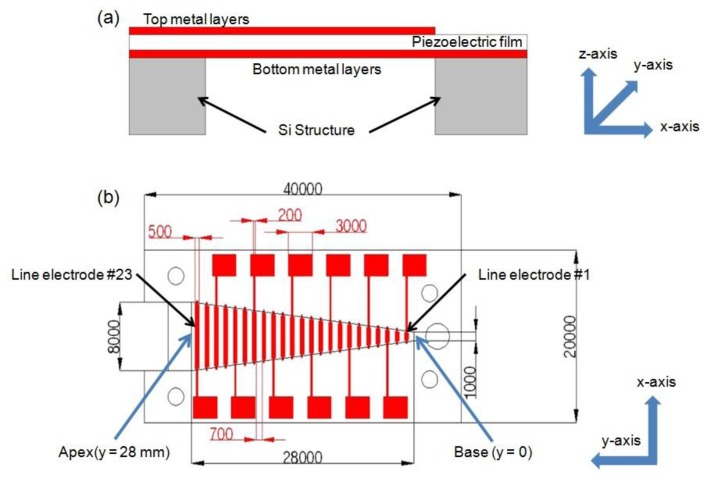
The schematic of artificial basilar membrane device from: (**a**) side view and (**b**) top view with dimensions in μm.

**Figure 2.**
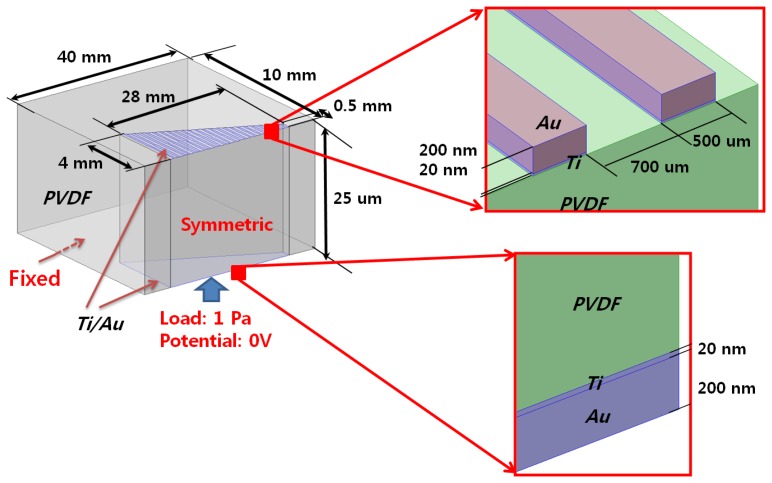
Finite element model geometry and boundary/load conditions.

Characteristics of a membrane, such as its thickness, width, stiffness and residual stress, determine its local resonance frequency and a membrane of varying characteristic along the y-axis has various local resonance frequencies at different locations along this axis. With increasing width, local resonance frequency decreases gradually. Piezoelectric film generates electrical output voltage in response to applied mechanical stress and strain. Each specific location of a membrane vibrates with relatively large amplitude at its local resonant frequency, thus generates larger electrical signal output on the resonating position than those on non-resonating positions. The schematic and dimensions of McPAS are shown in Figures 1 and 2.

It consists of a piezoelectric film layer, 23 discrete thin metal line electrodes (#1–#23) distributed evenly along the y-axis on the top of the film, and a silicon (Si) structure with a trapezoid opening supporting the film layer. The trapezoid height in y-axis is 28 mm and the short base (or base) and long base (or apex) of the trapezoid opening in the x-axis are 1 mm and 8 mm, respectively. The width (along the y-axis) of each metal line electrode is 500 μm and the length (along the x-axis) is the same as width of the opening at each position. The distance between neighboring electrodes is 700 μm. Twelve out of 23 line electrodes are connected to electrical pads for electrical signal measurement and additional thin metal layers on the bottom of the film are constructed as a common electrode.

To evaluate the designed McPAS, multiphysics finite element analysis (FEA) was carried out with the COMSOL Multiphysics^®^ software (COMSOL, Inc. Palo Alto, CA, USA). The FEA included both mechanical vibratory simulation and piezoelectric analysis with acoustic pressure load of various frequencies applied on the bottom of the membrane. The piezoelectric material properties of PVDF film reported by Tarn *et al.* [11] were used for the FEA. Detailed geometry and boundary/load conditions are shown in Figure 2.

**Figure 3.**
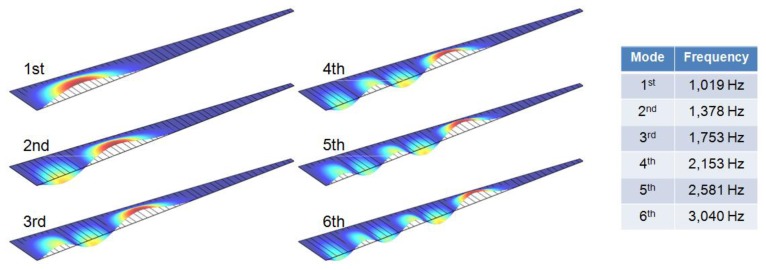
Resonance peak simulation results with mode analysis (1st–6th).

A finite element model was constructed to be half of the whole McPAS design (40 mm × 10 mm) and symmetric boundary conditions were applied to reconstruct the whole design simulation. Gold (Au) electrodes are present on both sides of the PVDF membrane for piezoelectric output. On the bottom nodes of the movable area (trapezoid shape), 1 Pa (94 dB SPL) sinusoidal pressure load was applied with potential condition of 0 V. A mode analysis of the McPAS model realized with COMSOL Multiphysics^®^ was carried out up to sixth mode (Figure 3). The colors and deformation indicate relative displacement of each point on the membrane. The local resonant position, which has maximum displacement (red color), changes gradually from apex area (wide width) to base (narrow width) area as mode number increases in a similar fashion as reported previously [7,10]. The frequency separation simulations in terms of vibratory behavior and piezoelectric signal output of the McPAS were also carried out and compared with experimental data (Figures 9, 10 and 11).

### 2.2. Fabrication

**Figure 4.**
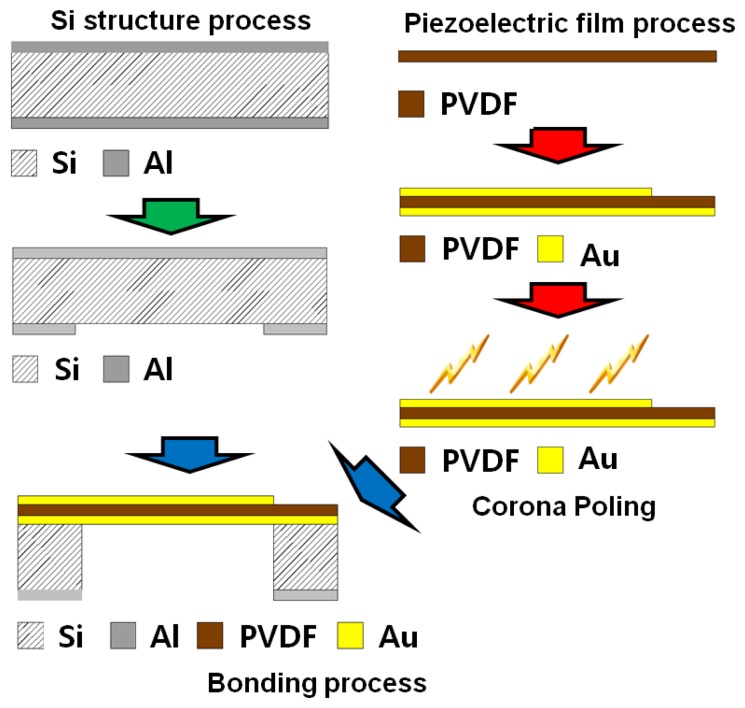
Schematic of the McPAS fabrication process.

**Figure 5.**
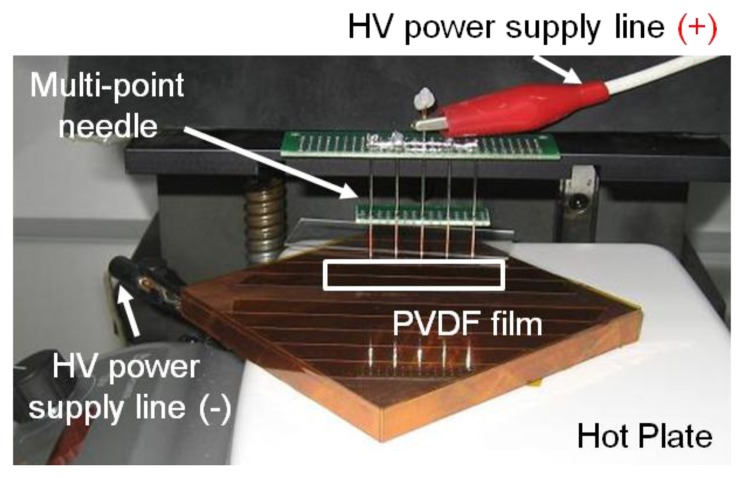
Corona-poling setup to enhance the piezoelectricity of PVDF film.

The fabrication steps can be divided into three phases: fabrication of the Si structure, piezoelectric film process and bonding process (Figure 4). The piezoelectric film used in this research is a polyvinylidene difluoride (PVDF) film of 25.4 μm thickness (Kynar^®^ Film, Professional Plastics, Singapore). First, a 300 nm thick aluminum (Al) masking layer was deposited on the front side of a Si wafer. Positive photoresist (AZ 1512, AZ Electronic Materials (Korea), Anseong, South Korea) was spin-coated and cured on the front side and a trapezoid opening was defined through ultra-violet (UV) light exposure and development. The exposed Al layer was etched first and residual photoresist was stripped away. Lift-off resist, photoresist and Al passivation layers were deposited in series on the bottom side of the substrate as masking layers. Finally, the trapezoid opening was realized with through-Si dry etching. The passivation layers were stripped after etching process. Second, 20 nm/200 nm thick titanium (Ti)/gold (Au) layers were deposited with a shadow mask on one side of a PVDF film with e-beam evaporator (KVET-C500200, Korea Vacuum Tech., Gimpo, South Korea) at the National NanoFab Center (NNFC, Daejeon, South Korea). The shadow mask was made of stainless steel and has openings for 23 line electrodes and 12 electrical pads. Additional thin metal deposition was carried out without a mask on the other side of the piezoelectric film. To enhance its piezoelectric property, a corona-poling process was followed on the processed PVDF film. The film was heated to 80 °C on a copper (Cu) block placed on a hot plate. After turning off the hot plate, high electric field was applied between the Cu block and needles positioned over the film for 60 min. The applied voltage was 7.5 kV and the distance between the Cu block and needles was 20 mm (Figure 5). The piezoelectric constant of the poled piezoelectric film was measured with PiezoMeter System (PM300, Piezotest, London, UK). The processed piezoelectric film was pre-stretched on a bonding pad and bonded onto the Si substrate with a trapezoid opening with the bottom side of the film facing the Si substrate.

### 2.3. Experimental Setup

**Figure 6.**
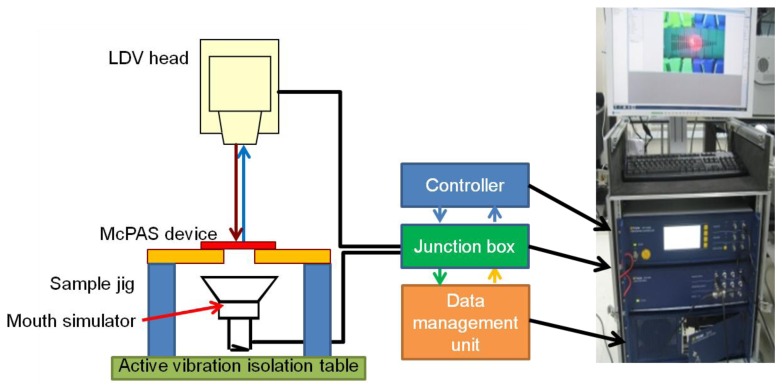
Schematic of experimental setup for vibratory property measurement of McPAS and picture of LDV controller system.

The vibratory properties of the developed McPAS were characterized with the experimental setup shown in Figure 6. The fabricated McPAS was fastened to a sample jig which in turn was securely bolted to an active vibration isolation table to minimize the effects of any external vibration. A mouth simulator (4227, B&K, Nærum, Denmark) was placed under the device to provide the acoustic signal. A scanning LDV head (PSV-I-400 LR and OFV-505, Polytec, Waldronn, Germany) was placed above the device to measure the velocity of the membrane in the z-axis while it vibrates. The central points of 23 line electrodes were chosen as the measurement points. The junction box of the LDV system is equipped with a function generator to drive the mouth simulator and input/output ports to connect to the controller and scanning LDV head. The data management unit can process the measured data to generate analysis results such as displacement, coherence, input/output transfer function of the device under test. The shape of electrical signal used to drive the mouth simulator was a periodic chirp over the frequency range of 3.125 Hz–20,000 Hz in 3.125 Hz steps. The measurement of membrane displacement at each measurement point was repeated 20 times to obtain an average for further analysis. Duration of each measurement was 320 ms.

**Figure 7.**
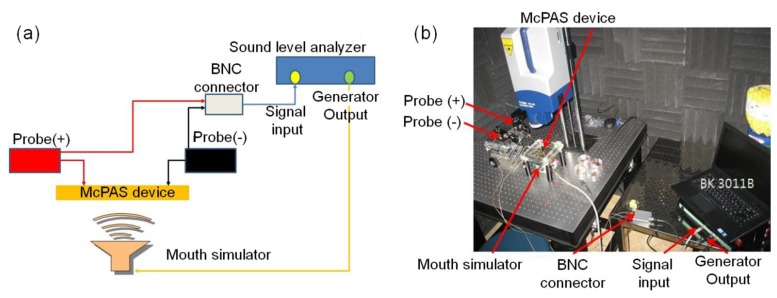
Schematic and picture of experimental setup for piezoelectric signal measurement.

The experimental setup for measuring the piezoelectric signal output is shown in Figure 7. The piezoelectric signal from each electrical pad on the PVDF membrane was measured with a sound level analyzer (BK3011, BaKo, Suwon, South Korea).

The sound level analyzer can provide a sinusoidal signal to a mouth simulator and receive the electrical signal output from an acoustic sensor device. Measured data can be analyzed and displayed in terms of sensitivity of acoustic device versus frequency of applied signal over the pre-defined frequency range. The sound level analyzer was calibrated with a reference microphone (2671, B&K) before each experiment to ensure a constant sound pressure level was applied to the McPAS over the pre-defined frequency range. The piezoelectric signal output was measured differentially as the negative probe (Probe(−)) was connected with the common electrode and the positive probe (Probe(+)) was in contact with one of 12 electrical pads.

## 3. Experimental Results

**Figure 8.**
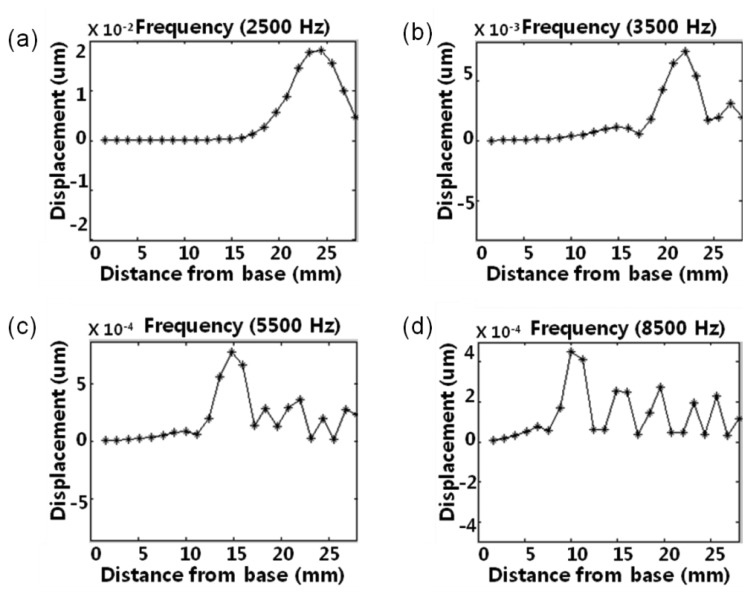
The local resonant response of the McPAS membrane with sound sources of (**a**) 2.5 kHz, (**b**) 3.5 kHz, (**c**) 5.5 kHz, and (**d**) 8.5 kHz.

The local resonant behaviors of the membrane were measured at the central points of 23 line electrodes on the membrane using the scanning LDV system. The McPAS has a gradually varying membrane width from 1 mm at y = 0 mm to 8 mm at y = 28 mm. The measured local resonant responses of the membrane are shown in Figure 8. With a sound source of 2.5 kHz, the local resonance took place near the apex area (y = 23.7 mm) and there was one maximum. However, the sound sources of 3.5 kHz, 5.5 kHz and 8.5 kHz created local resonant responses near y = 21.3 mm, y = 14.1 mm and y = 9.3 mm, respectively. As the frequency of sound source increased, the position showing the local resonant response moved closer to the base (y = 0 mm).

**Figure 9.**
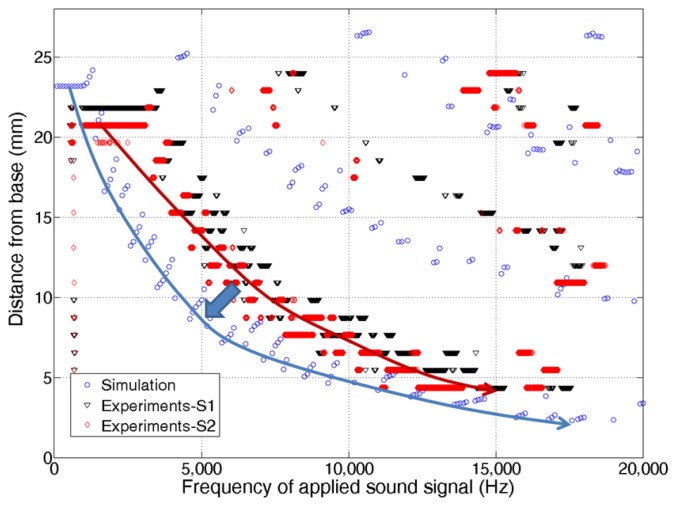
Frequency separation of the McPAS device under mechanical vibration.

To characterize its frequency response more in detail, the measured local resonances were further analyzed and compared with simulation results. For simulation, acoustic pressure (or load) over the frequency range of 100 Hz–25,000 Hz with 100 Hz steps was applied on the bottom side of membrane of the developed model (Figure 2) as sound source. Figure 9 shows experimental data of two McPASs (S1, S2) and FEA simulation results displaying the y-position of the largest resonant peak along the centerline at each applied sound source with different frequencies. The position of maximum displacement changes gradually from the apex area to base area as the frequency of input signals varies from 2.5 kHz to 13.5 kHz, demonstrating the capability of the McPAS to act as an artificial basilar membrane or frequency analyzer. Several test samples were fabricated and characterized. There were small discrepancies in the experimental data between test samples. Both simulation and measurement results show quite similar frequency response patterns, but there are discrepancies between the positions of largest resonant peaks. These discrepancies seem to arise from surface tension present on the membrane which is created during the fabrication process.

**Figure 10.**
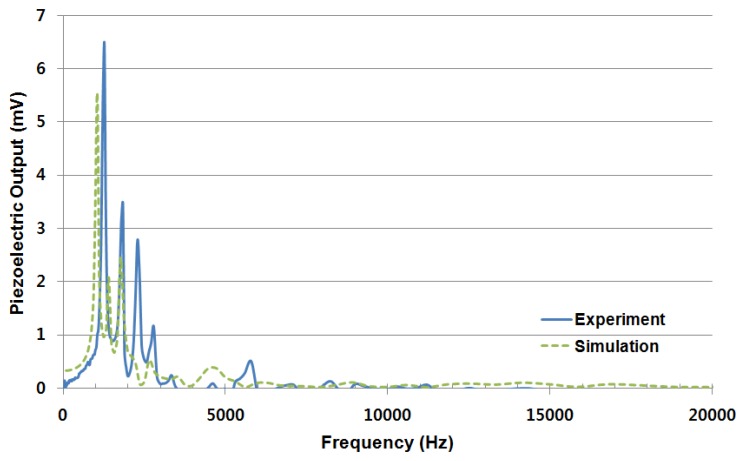
Piezoelectric signal output of line electrode #3.

The corona-poling process was intended to enhance the piezoelectricity of the piezoelectric film. The piezoelectric constant of the poled piezoelectric film was measured with the Piezotest PM300 PiezoMeter System. The d33 value of piezoelectric constant was below 0.22 pC/N without any poling process and increased to 1.60–4.05 pC/N after the corona-poling process. Piezoelectric signal measurement from the electrodes on the McPAS while exposed to external sound source of 1 Pa (94 dBSPL) over the frequency range of 100 Hz and 20,000 Hz were conducted using a sound level analyzer and mouth simulator as shown in Figure 7. Figure 10 shows the resulting piezoelectric signal output in peak-to-peak voltage (Vpp) from line electrode #3 having maximum value of 6.33 mVpp at 1,250 Hz (−53 dBV/Pa, 0 dBV = 1 Vrms). Like the vibratory results, there are slight frequency offsets between the experimental and simulation results, but the overall patterns of the measured results are in good agreement with the simulation results.

**Figure 11.**
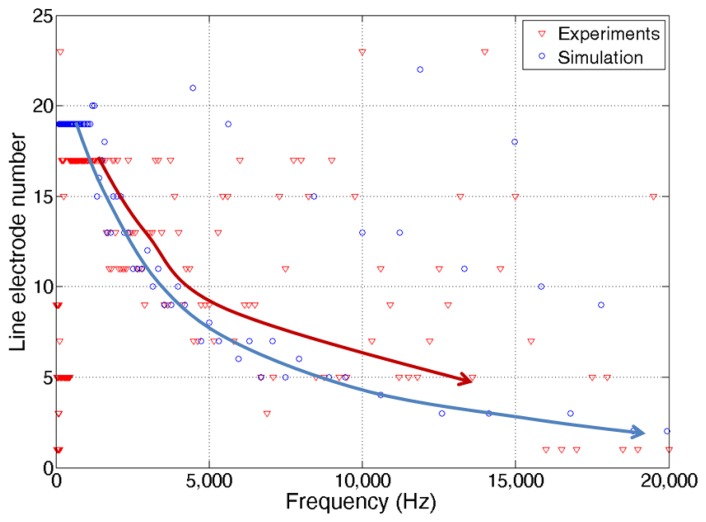
Frequency separation of the McPAS device in the piezoelectric signals.

Additional experiments were carried out to determine the position or line electrode number of the maximum piezoelectric signal output at each input sound frequency (Figure 11). The experiment data show that at lower input sound frequency, line electrodes near the apex (high line electrode number) generated the maximum piezoelectric output while at higher input sound frequency, line electrodes near the base (low line electrode number) responded the most. The piezoelectric signal output simulation results are almost same as those of vibratory displacement. However, the measured maximum output positions are more scattered compared to those of vibratory displacement on the graph. Non-uniform piezoelectric constant measured over the membrane after corona-poling (1.60–4.05 pC/N) and the lesser number of measurement points (23 for displacement and 12 for piezoelectric signal) seem to affect the piezoelectric signal output performance.

## 4. Conclusions/Outlook

In this study, we have presented the development of an artificial basilar membrane device mimicking the function of human cochlea, including its design, FEA verification, fabrication and characterization. The developed sensor can separate the acoustic signals based on their frequency and convert membrane vibrations into electrical signals. Multiphysics finite element analysis of both the mechanical vibration and piezoelectric characteristics was carried out to verify the function of the McPAS design as an artificial basilar membrane and the McPAS was fabricated with microfabrication techniques. The vibratory characteristic was measured using a scanning LDV system and piezoelectric properties were measured with a sound level analyzer. The experimental vibratory and piezoelectric property results follow the simulation results and both results demonstrated that the developed multi-channel piezoelectric acoustic sensor (McPAS) can separate the acoustic signals spatially and generate the corresponding piezoelectric signals without an external power source. Higher frequency signals resonated at a location closer to the base. The frequency separation range for the developed McPAS was 2.5 kHz–13.5 kHz and the maximum electrical signal output from an electrode upon acoustic signal input of 94 dBSPL was 6.33 mVpp. To enhance the performance of the McPAS as an artificial basilar membrane, aspects of the fabrication processes such as detailed control of surface tension and an optimized corona-poling process will be investigated further. Also, with the developed simulation model and comparison of the data with experimental results, design optimization for miniaturizing a McPAS capable of frequency separation over the human audible frequency range and implantable inside the mastoid cavity will follow.

The presented McPAS is a step towards development of total implantable cochlear system requiring much less power, exhibiting more natural performance in sound conversion compared to conventional cochlear implants and thus contributing to the enhancement the quality of life for those suffering from hearing loss.
